# Multiplex Real-Time qPCR Assay for Simultaneous and Sensitive Detection of Phytoplasmas in Sesame Plants and Insect Vectors

**DOI:** 10.1371/journal.pone.0155891

**Published:** 2016-05-19

**Authors:** Cengiz Ikten, Rustem Ustun, Mursel Catal, Engin Yol, Bulent Uzun

**Affiliations:** 1 Department of Plant Protection, Faculty of Agriculture, Akdeniz University, TR-07058, Antalya, Turkey; 2 Department of Field Crops, Faculty of Agriculture, Akdeniz University, TR-07058, Antalya, Turkey; Istituto Biologia e Biotecnologia Agraria IBBA, ITALY

## Abstract

Phyllody, a destructive and economically important disease worldwide caused by phytoplasma infections, is characterized by the abnormal development of floral structures into stunted leafy parts and contributes to serious losses in crop plants, including sesame (*Sesamum indicum* L.). Accurate identification, differentiation, and quantification of phyllody-causing phytoplasmas are essential for effective management of this plant disease and for selection of resistant sesame varieties. In this study, a diagnostic multiplex qPCR assay was developed using TaqMan^®^ chemistry based on detection of the 16S ribosomal RNA gene of phytoplasmas and the 18S ribosomal gene of sesame. Phytoplasma and sesame specific primers and probes labeled with different fluorescent dyes were used for simultaneous amplification of 16SrII and 16SrIX phytoplasmas in a single tube. The multiplex real-time qPCR assay allowed accurate detection, differentiation, and quantification of 16SrII and 16SrIX groups in 109 sesame plant and 92 insect vector samples tested. The assay was found to have a detection sensitivity of 1.8 x 10^2^ and 1.6 x 10^2^ DNA copies for absolute quantification of 16SrII and 16SrIX group phytoplasmas, respectively. Relative quantification was effective and reliable for determination of phyllody phytoplasma DNA amounts normalized to sesame DNA in infected plant tissues. The development of this qPCR assay provides a method for the rapid measurement of infection loads to identify resistance levels of sesame genotypes against phyllody phytoplasma disease.

## Introduction

Sesame (*Sesamum indicum* L.) is one of the first oilseed plants to be used for many different purposes [1,2]. It has one of the highest oil contents among oil crops [3,4] which predominantly contains oleic and linoleic acids [5,6]. Sesame seeds are used as raw food as well as in confectionery and bakery products. Sesame grows well in tropical and subtropical climates and can be cultivated in mixed stands with diverse crops or in areas without rainfall or irrigation [7]. Despite its long domestication history and health benefits, the production of sesame has been hampered by low seed yield, disease susceptibility, non-mechanized harvesting, seed shattering, indeterminate growth habit, and stiff competition from exotic oil crops [8]. In the last two decades, symptoms associated with phytoplasma disease, phyllody, strongly impacted the decline of cultivated sesame in India, Iran, Iraq, Israel, Burma, Turkey, Oman, Sudan, Nigeria, Tanzania, Pakistan, Ethiopia, Thailand, Uganda, Upper Volta, Vietnam, and Mexico [9], with a disease incidence between 12 to 80% [10–13]. Symptomatically, the disease causes stunted growth and alteration of the floral parts into leafy structures bearing no capsules and seeds, resulting in significant economic losses [14].

Phytoplasmas are non-helical obligate parasites that belong to the prokaryotic class Mollicutes, and are transmitted by sap-feeding insects and vegetative plant propagation materials [15–18]. Diagnosis of phytoplasma pathogens has proven difficult because they cannot be cultured *in vitro* [19–21]. Before the advent of molecular techniques, disease symptoms, insect or dodder graft transmission to host plants, and electron microscopy were used for detection of phytoplasmas [22]. Following the initial cloning of phytoplasma DNA [23], nucleic acid-based applications have been developed for the detection and identification of phytoplasmas in plants and vectors. Polymerase chain reaction (PCR) amplification of 16S rRNA with either phytoplasma species-specific primers or phytoplasma group-specific primers has been preferred widely for molecular diagnostics [20,24]. In particular, the nested-PCR approach has allowed the establishment of a specific detection of different species and strains of phytoplasmas [25]. Until recently, 28 phytoplasma groups and 50 subgroups have been identified by PCR techniques [26]. Although PCR methods have been used widely in phytoplasma identification and classification, they have many disadvantages, including time-consuming multi-step analyses with limited sensitivity, requirement for intense labor, and the risk of cross contamination during manipulation [27,28].

Real-time quantitative PCR (qPCR) is a powerful alternative method for phytoplasma detection and quantification because it offers elevated detection sensitivity, short analysis time, and high automation capability [29]. Real-time qPCR reduces the chance of contamination in a closed-tube system, does not require two round reactions, and sensitive fluorescence detection tools eliminate the need to analyze reaction products by gel electrophoresis [30]. Furthermore, the high sensitivity of this method allows for quantitative description of all phases of phytoplasma infection [31] when labeling systems are used for the DNA amplicons [25]. Real-time qPCR assays have been developed for individual or group specific detection and quantification of many phytoplasmas [18,25,32–35] based on the 16S rRNA [36] and 23S rRNA gene sequences [37].

Sesame phyllody, an economically important disease of sesame plants, is a serious threat in regions where peanut witches’ broom (16SrII) [9,12,38,39], pigeon pea witches’ broom (16SrIX) [40], aster yellows (16SrI) [41,42], and clover proliferation (16SrVI) [14] phytoplasmas are present. Although four different phytoplasma groups have been reported in sesame so far, the majority (approximately 77%) of identified sesame phytoplasmas have been known to belong to the 16SrII and 16SrIX groups [43]. Therefore, quantification of infection levels and enhanced detection in a large number of samples of 16SrII and 16SrIX group phytoplasmas are important universal problems in the global struggle against sesame phyllody disease. The goals of this study were to develop a qPCR assay for detection, differentiation, and quantification of 16SrII and 16SrIX group phytoplasma pathogens in sesame plants and insect vectors and to employ this quantitative assay for determination of phytoplasma amounts in infected plant and insect tissues.

## Materials and Methods

### Ethics Statement

Permissions from the owners of sesame fields for sampling were obtained. No endangered or protected species were involved in the study.

### Plant and insect samples

Plant and insect samples were collected from naturally infected sesame fields in the Aksu, Aspendos, Batem, Belkıs, Beşkonak, Boğazkent, Bozova, Cumalı, Denizyaka, Gündoğdu, Kadriye, Kocayatak, Kovanlık, and Taşağıl locations of Antalya province from June to September 2011 through 2014 (Table 1). Additionally, samples for positive and negative controls were obtained from sesame plants grown in cages with and without vectors in a greenhouse located at Akdeniz University Campus. Sesame plants in fields were observed visually for phyllody symptoms on foliage, flowers, and capsules and both symptomatic and asymptomatic plants were sampled. Vector insects were captured using a hand-held vacuum tool in the early morning hours between 8:00 and 10:00 am. Samples collected were stored at -20°C until use. Since *Orosius orientalis* was identified as the only insect vector of phyllody phytoplasmas in our previous study [39], the qPCR assay was focused on this leafhopper. The field collected samples were subjected to direct and nested PCR for further confirmation of phytoplasma presence [39,44,45].

**Table 1:** Multiplex qPCR detection of 16SrII and 16SrIX group phytoplasmas in plant and insect samples collected from different locations of Antalya province in Turkey.

Locations	Plant				Insect			
	Total	16SrII	16SrIX	16SrII+16SrIX	Total	16SrII	16SrIX	16SrII+16SrIX
Aksu	7	7	0	0	6	0	0	0
Aspendos	2	1	0	1	0	0	0	0
Batem	14	8	6	0	12	2	0	0
Belkıs	4	4	0	0	0	0	0	0
Beşkonak	0	0	0	0	5	0	0	0
Boğazkent	15	14	1	0	15	1	0	0
Bozova	0	0	0	0	1	0	0	0
Cumalı	0	0	0	0	9	0	0	0
Denizyaka	30	25	0	2	17	2	0	0
Gündoğdu	6	5	0	1	0	0	0	0
Kadriye	1	1	0	0	0	0	0	0
Kocayatak	0	0	0	0	6	1	0	0
Kovanlık	23	11	7	0	11	1	1	0
Taşağıl	3	0	0	0	7	2	0	0
Greenhouse	4	0	0	0	3	2	0	0
**Total**	**109**	**76**	**14**	**4**	**92**	**11**	**1**	**0**

### DNA extraction

Total DNA from samples of both sesame plant and vector insects was extracted by the CTAB method [46]. A 100 mg sample of fresh sesame leaf tissue or one whole insect was used to extract DNA from field-collected samples. Agarose gel electrophoresis and spectrophotometry were used to determine the quality and quantity of extracted DNA samples. Total extracted DNA was dissolved in Milli-Q PCR-grade water and stored at -20°C until use.

### Primer and probe design

**Fig 1:**
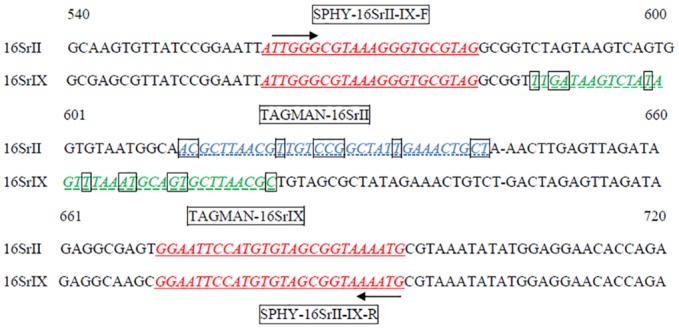
Sequence alignment of the 16S ribosomal gene region used for amplification and specific detection of sesame 16Sr group II and IX phytoplasmas. Sequences of the primers and probes designed in this study are shown in underlined and italic letters, respectively. Forward primer SPHY-16SrII-IX-F (upper arrow, red), probe 16SrII (dotted line, blue) and 16SrIX (solid line, green), and reverse primer SPHY-16SrII-IX-R (lower arrow, red) are noted. The sequence differences between 16SrII and 16SrIX in the probe regions were shown in boxed letters.

Primers and probes designed in this study are listed in Table 2. The primer pair SPHY-16SrII-IX-F/SPHY-16SrII-IX-R was designed from the 16S ribosomal RNA gene sequence for specific amplification of 16Sr group II and IX as well as other 16Sr group phytoplasmas. The probes 16SrII and 16SrIX were designed from the sequences between these primer pairs for specific detection of 16Sr group II and group IX phytoplasmas, respectively, in TaqMan^®^ qPCR assay. Representative sequences of 16Sr groups I through XIV [47,48,49] (S1 Fig) including sesame phyllody 16Sr groups II and IX [39,40] were aligned to design the primer and probes (Fig 1). The sequences of all phytoplasma groups available in the National Center for Biotechnology Information (NCBI) GenBank database were also included in the alignments. The 16Sr sequences were aligned using SeqMan and MegAlign in the Lasergene software package (DNASTAR Inc., Madison, WI). The primer pair SEPL18SDNA-F/SEPL18SDNA-R and the probe SESAM-TaqMan^®^ were designed from the18S ribosomal DNA sequence (AJ236041) to detect sesame plant DNA in qPCR. This sesame DNA specific primer pair and probe were developed to serve as an endogenous control to normalize the DNA quantities and to allow for relative quantification of the phytoplasmas in infected plant tissue. The plant primer pair and probe combination were also used to verify the quality of extracted DNA and the absence of PCR inhibitors. Primer Express software (Applied Biosystems, Foster City, CA) was used to design qPCR primers and probes. They were synthesized by Metabion International AG (Semmelweisstrasse, Germany).

**Table 2:** Primers and TaqMan^®^ probes designed for multiplex qPCR detection of 16SrII and 16IX phytoplasmas and sesame DNA.

Primers/Probes	Sequence (5′-3′)	Tm	GC (%)	Amplicon (bp)
SPHY-16SrII-IX-F^1^	ATTGGGCGTAAAGGGTGCGTAG	59	55	136
SPHY-16SrII-IX-R^1^	CATTTTACCGCTACACATGGAATTCC	59	42	
TaqMan^®^16SrII^2^	LC Red 640-ACGCTTAACGTTGTCCGGCTATTGAAACTGCT-BHQ-2	68	47	
TaqMan^®^16SrIX^3^	Texas Red-TTGATAAGTCTATAGTTTAAATGCAGTGCTTAACGC-BHQ-2	63	33	
SEPL-18SDNA-F^4^	CGCGGAAGTTTGAGGCAATA	55	50	103
SEPL-18SDNA-R^4^	CTGTCGGCCAAGGCTATAGACT	55	55	
TaqMan^®^SESAME^5^	HEX-TAGATGTTCTGGGCCGCACGCG-TAMRA	66	64	

^1^Phytoplasma specific universal primers

^2^16SrII group phytoplasma specific hybridization probe

^3^16SrIX group phytoplasma specific hybridization probe

^4^Sesame housekeeping gene primers

^5^Sesame housekeeping gene specific hybridization probe

### Specificity of qPCR

Specificity of qPCR assay for 16SrII and 16SrIX group phytoplasmas was determined by testing against DNA from 4 phytoplasma samples representing 4 different 16Sr groups. The samples of phytoplasma strains PPT (Potato, purple top/*Candidatus phytoplasma asteris*-16SrI-C), MA (Daisy, yellow/*Candidatus phytoplasma pruni*-16SrIII-B), ASHY2 (Ash, yellow/*Candidatus phytoplasma fraxini*-16SrVII-A), and AP15 (Apple proliferation/*Candidatus phytoplasma mali*-16SrX-A) were kindly provided by Dr. Assunta Bertaccini of the University of Bologna in Italy. Extracted DNA from phytoplasma samples was amplified with universal phytoplasma primers by nested PCR [44,45] to verify suitability for qPCR assays. Aliquots of 2 μl extracted DNA (10 ng per reaction) were run in triplicate for each qPCR assay.

### TaqMan^®^ qPCR development

qPCR amplifications were carried out in single optical tubes using the Rotor-Gene Q 5plex HRM Platform (Qiagen, Hilden Germany). qPCR was performed in 20 μl reaction volumes, containing 2XTaqMan PCR master mix (Fermentas, Lithuania), 300 nM forward and reverse primers, 150 nM 16SrII LC Red, 16SrIX Texas Red, and Sesame 18S-Hex labeled probes, and 2 μl of template DNA (10 ng per reaction). The thermal cycling protocol consisted of an initial denaturation at 95°C for 10 min, followed by 45 cycles at 95°C for 15 sec and at 60°C for 1 min. Cycle threshold (Ct) values were calculated and analyzed with the Rotorgene Q series software version 1.7 (Qiagen Inc, Valencia, CA, USA).

qPCR assays were used to determine both absolute and relative quantities of 16SrII and 16SrIX group phytoplasmas in sesame plant samples and absolute quantities only in insect samples. DNA extracted from sesame phyllody plant and insect samples was added to a multiplex qPCR reaction mix and amplified to determine the amounts of each phytoplasma in field samples. Negative control (uninfected sesame checked by nested PCR), and non-template control (PCR water) were included in each independent run. Standards were prepared by amplifying 16S rRNA sequences of 16SrII and 16SrIX group sesame phyllody phytoplasmas directly with the primer pair Fu5/Ru3 from DNA extracted from sesame samples infected with only the respective 16Sr group phyllody phytoplasmas in conventional PCR [44,45]. Amplification products of approximately 880 bp were purified with a QIAquick^®^ PCR purification kit (Qiagen, Hilden Germany) and quantified using a spectrophotometer. The copy number of PCR amplicons was calculated using the following formula: Copy number = (Amplicon amount x Avogadro's number) / (Amplicon size in bp × 650 x 1 x 10^9^) [50]. A subset of 10-fold serial dilutions of DNA from each 16SrII and 16SrIX group, ranging from 1.8 × 10^8^ to 1.8 × 10^2^ and 1.6 × 10^8^ to 1.6 × 10^2^ copies per reaction, respectively, was qPCR-amplified in triplicate to generate a standard curve for each phytoplasma group. qPCR assays were also performed to determine whether PCR inhibitors were present in DNA extracted from sesame plant tissues. Each dilution series of 16SrII and 16SrIX group DNA used for construction of phytoplasma standard curves was added separately to 2 μl (10 ng per reaction) sesame DNA from healthy tissue to produce the same final concentrations in each reaction and simulate proportions of naturally infected sesame tissue. Standard curves were also created from amplification of these samples containing 16SrII or 16SrIX group DNA and sesame DNA. Absolute quantities of 16SrII and 16SrIX phytoplasma DNA in infected sesame plant and insect tissues were calculated based on standard curves using comparative cycle threshold (Ct) values of sesame field test samples.

To measure relative quantities of 16SrII and 16SrIX group phytoplasma DNA in sesame tissues, relative qPCR using the comparative Ct method was used as described in Rotor-Gene Q series user bulletins (Qiagen, Hilden, Germany). Ten ng of total DNA extracted from field collected sesame tissues was simultaneously amplified with both phytoplasma specific and endogenous control primers (18S rDNA) in the same tubes. The lowest detectable dilutions of 16SrII and 16SrIX determined by inhibition assays were designated as calibrators for phytoplasma group quantification experiments. The quantities of calibrators were regarded as 1 and the quantities of all the other field samples were expressed as n-fold difference relative to the calibrators. Absolute quantities calculated from standard curves were omitted in relative quantification because sample quantities were divided by the calibrator quantity. Quantities of 16SrII and 16SrIX group DNA normalized to the endogenous control sesame DNA and relative to the calibrators were either calculated automatically by the software or manually [51].

## Results

### Specificity of qPCR

Preliminary real-time PCR assay with SYBR^®^ Green (Thermo Fisher Scientific, Carlsbad, CA) revealed that the primer pair SPHY-16SrII-IX-F/SPHY-16SrII-IX-R amplified a 136 bp PCR product from all 16Sr group phytoplasma sequences tested (Table 2, S2 Fig). All the sequences of the 16Sr groups tested in this study and of those from GenBank database shared 100% homology at primer sites. TaqMan^®^ probes 16SrII and 16SrIX were designed from the most variable region between the primers to hybridize specifically target DNA of 16Sr groups II and IX, respectively (S1 Fig). To provide specificity, the 16SrII probe sequence differed from the 16SrIX group sequence by 9 bp, and the 16SrIX probe sequence differed from 16SrII group sequence by 10 bp (Fig 1). The primer pair SPHY-16SrII-IX-F/SPHY-16SrII-IX-R in combination with probe 16SrII detected only phytoplasmas of group II and with probe 16SrIX only phytoplasmas of group IX in multiplex qPCR (S3 Fig). The primers consistently produced Ct values of ten and higher with TaqMan probes of both 16SrII and 16SrIX groups and amplified only the target DNA of corresponding phytoplasma group. The primers with each probe separately or in combination did not hybridize to the DNA from any of the 4 phytoplasma strains PPT (16SrI-C), MA (16SrIII-B), ASHY2 (16SrVII-A), or AP15 (16SrX-A), representing the four major phytoplasma 16Sr groups. As expected, primers in combination with each probe did not yield Ct values for DNA from uninfected sesame plants (negative control) or non-template water (S3 Fig).

### Sensitivity of the assays and detection limits

**Fig 2:**
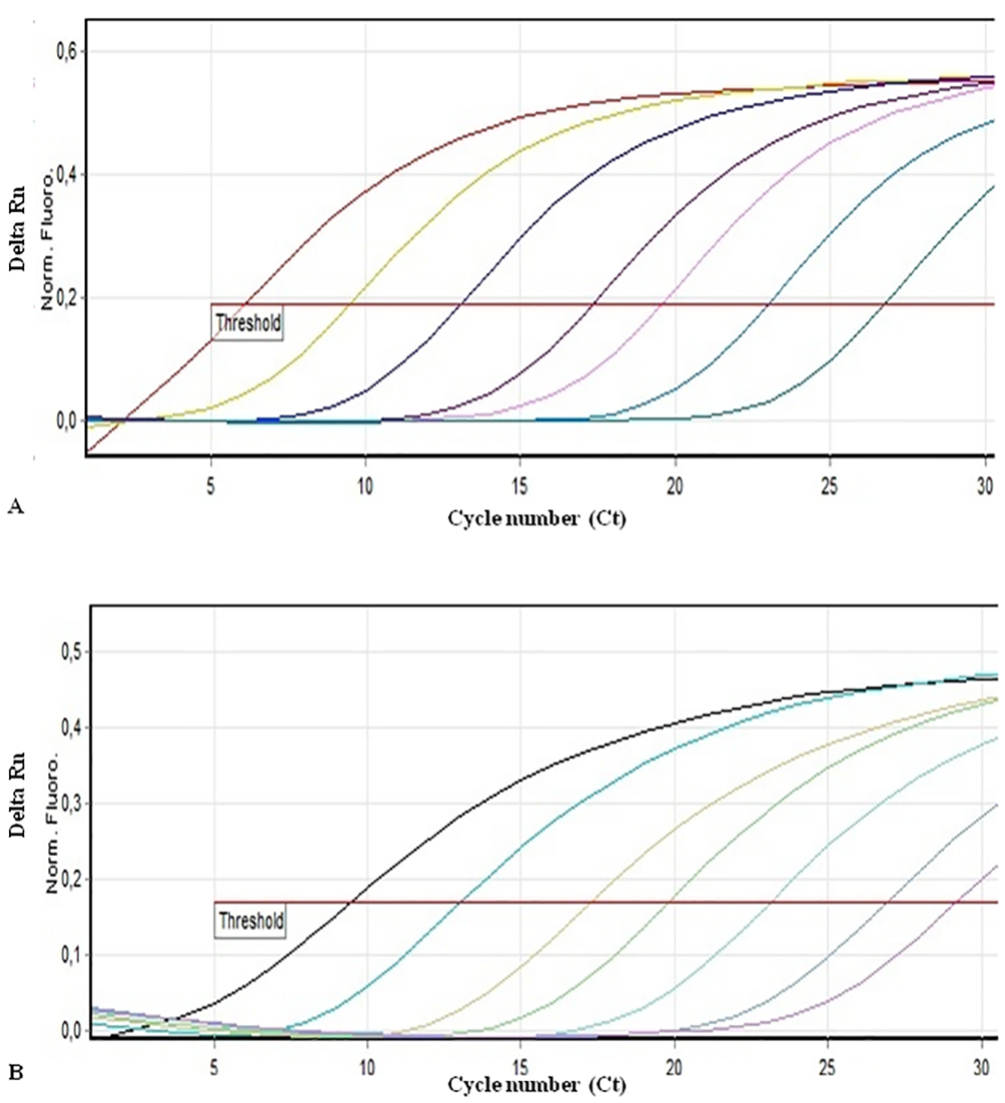
Multiplex qPCR amplification of seven 10-fold serial dilutions of 16SrII (A) and 16SrIX (B) group phytoplasma DNA (purified PCR products). The amplification mixture contained phytoplasma and sesame primers and probes. Each dilution series was mixed with a constant 10 ng (2 μl) of healthy sesame plant DNA in each reaction to determine whether PCR inhibitors were present. Each line represents one of the experiment made in triplicate. Delta Rn (ΔRn) values show the fluorescence emission accumulation normalized to the background emission. Cycle threshold (Ct) values indicate specific cycles at which ΔRn passes the threshold value.

Phytoplasma specific SPHY-16SrII-IX-F/R and sesame plant DNA SEPL-18SDNA-F/R primer pairs were used in various combinations with 16SrII, 16SrIX and sesame plant probes to amplify 7 serial dilutions (10-fold each) of 16SrII and 16SrIX purified PCR products, and sesame DNA to detect sensitivity limits of the qPCR assay (Fig 2). The initial amounts of 16SrII and 16SrIX DNA were approximately 1.8 × 10^8^ and 1.6 × 10^8^ copies per reaction, respectively. Amplification graphs and standard curves yielded reproducible and reliable Ct values in replicated assays. The primer pair SPHY-16SrII-IX-F/R was found to have a detection limit of 1.8–1.6 × 10^2^ phytoplasma cells per reaction when used with 16SrII and 16SrIX probes alone or in combination (Table 3). Linear relationships were identified when Ct values were plotted against the logs of starting quantity 16SrII or 16SrIX DNA. Furthermore, no cross signal amplification was observed from 16SrII DNA combined with the 16SrIX probe or vice versa, indicating that these probe qPCR assays were specific for the intended phytoplasma groups despite using highly concentrated templates from purified PCR products (Table 3).

**Table 3:** Slope, intercept, correlation coefficients (R^2^), and efficiencies (E) of qPCR assay.

Primer pair/combinations	Probe/combinations	DNA^1^	qPCR^2^	Slope	Intercept	R^2^	E
SPHY-16SrII-IX-F/R	16SrII	16SrII	16SrII	-3.348	30.505	0.98	0.99
SPHY-16SrII-IX-F/R	16SrII	16SrIX	NA				
SPHY-16SrII-IX-F/R	16SrIX	16SrIX	16SrIX	-3.429	30.309	0.99	0.98
SPHY-16SrII-IX-F/R	16SrIX	16SrII	NA				
SPHY-16SrII-IX-F/R	16SrII+16SrIX	16SrII	16SrII	-3.355	30.494	0.99	0.99
SPHY-16SrII-IX-F/R	16SrII+16SrIX	16SrIX	16SrIX	-3.342	29.568	0.99	0.99
SEPL-18SDNA-F/R	Sesame	16SrII	NA				
SEPL-18SDNA-F/R	Sesame	16SrIX	NA				
SEPL-18SDNA-F/R	Sesame	Sesame	Sesame	-3.366	26.469	0.98	0.99
SPHY-16SrII-IX-F/R-SEPL-18SDNA-F/R	16SrII+16SrIX+Sesame	16SrII	16SrII	-3.754	31.359	0.99	0.98
SPHY-16SrII-IX-F/R-SEPL-18SDNA-F/R	16SrII+16SrIX+Sesame	16SrIX	16SrIX	-3.510	31.388	0.98	0.98

^1^Indicates 16Sr group phytoplasma DNA that was serially diluted 7 times (10× each) and used in qPCR.

^2^Represents the amplified 16Sr group phytoplasma

NA: Not amplified

Internal control primer pair efficiently amplified sesame plant DNA through at least five orders of magnitude of 10-fold dilutions beginning with 1.0 × 10^4^ pg of DNA. Addition of sesame SEPL-18SDNA-F/R primers and the SESAME probe into phytoplasma primers and probes did not affect specificity. The slope, intercept, correlation coefficient, and efficiency values of standard curves for all assays are given in Table 3. Efficiencies of amplifications with 16Sr phytoplasma and internal control sesame primers and probes were close to one another as observed by the slopes of the standard curves.

### Accuracy of qPCR in inhibition assays

**Fig 3:**
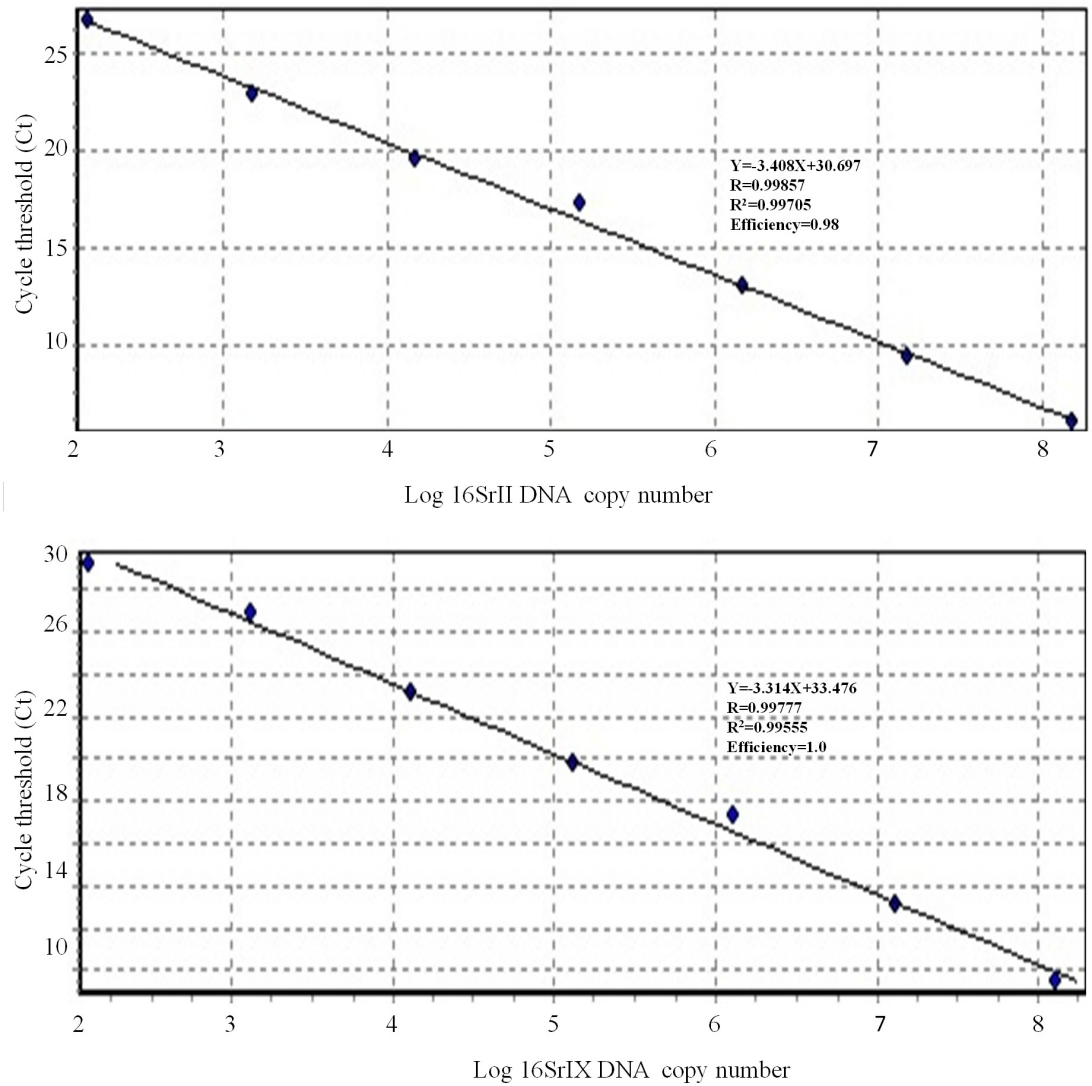
Standard curves generated from the multiplex qPCR amplification of seven 10-fold dilution series of 16SrII (A) and 16SrIX (B) group standards used to convert Ct values to the phytoplasma DNA copies present in the samples. The amplification mixture contained phytoplasma and sesame primers, as well as 16SrII, 16SrIX, and sesame probes. Each dilution series was added to 10 ng of uninfected sesame DNA per reaction as part of the inhibition experiments. Log_10_ values of the initial copies of phytoplasma DNA are plotted against corresponding Ct values.

A qPCR reaction combination of phytoplasmas and sesame primers and probes was used to amplify serial DNA dilutions of the target phytoplasma group (16SrII or 16SrIX) DNA in the presence of sesame plant DNA in inhibition tests. The multiplex assays successfully amplified the same seven 10-fold target 16SrII and/or 16SrIX DNA dilutions and resulted in consistent Ct values through 30 cycles (Fig 3). The standard curves generated by these 7 dilutions of 16SrII or 16SrIX standards mixed with sesame plant DNA correlated well with standard curves previously constructed by the same serial dilutions of the phytoplasmas DNA in molecular grade water (data not shown).

Detection limits of the multiplex qPCR assay for 16SrII and 16SrIX group phytoplasmas in the mixed phytoplasmas and sesame DNA were found to be 1.8 × 10^2^ and 1.6 × 10^2^ copies per reaction, respectively. Amplification efficiencies of the 16SrII (0.98) and 16SrIX (1.0) assay in the presence of sesame DNA (Fig 3) were found to be similar to those obtained from DNA dilutions in water. Similar linear relationships were found between the initial amounts of 16SrII or 16SrIX DNA mixed with sesame DNA and the Ct values obtained from standard curves using data from phytoplasma DNA in molecular grade water. The correlation coefficients of the standard curves created in the inhibition assay were 0.99705 for 16SrII and 0.99555 for the 16SrIX specific qPCR assays (Fig 3).

### Detection of phyllody phytoplasmas in sesame plants and insect vectors

DNA extracts from 109 plant and 92 insect samples collected from 15 different locations were tested for the presence of sesame phytoplasmas in multiplex qPCR developed in this study (Table 1). Amplifications were carried out in the same reaction tubes that contained phytoplasma and sesame primers and 16SrII, 16SrIX, and sesame probes. The results of the detection assays showed that 76 plant samples were infected with 16SrII and 14 with 16SrIX group phytoplasmas. Four plant samples were shown to be co-infected with both 16SrII and 16SrIX phytoplasmas, and 15 symptomless samples were free of phyllody phytoplasmas (no infections detected). Of 92 insect samples tested, only 11 were infected with 16SrII and one with 16SrIX phytoplasmas. No phytoplasmas were detected in 80 insect samples.

### Quantification of sesame phyllody phytoplasmas

Absolute and relative quantities of 16SrII and 16SrIX phytoplasma DNAs were determined for the 94 samples from which sesame phytoplasmas were detected (Table 4). The samples from Gundogdu district (1.75 × 10^7^), had the highest and Bogazkent (1.12 × 10^6^) lowest absolute quantities (copies) of 16SrII DNA per mg of plant tissue. In relative quantification, the highest and lowest amounts of 16SrII phytoplasma DNA were from the Gundogdu and Belkıs district samples (Table 4). Absolute and relative quantities of 16SrIX phytoplasma DNA were much lower than 16SrII DNA quantities in the samples. The samples from Batem (2.31 × 10^6^) contained the highest amounts of 16SrIX phytoplasma per mg of sesame tissue.

**Table 4:** Absolute and relative quantities of 16SrII and 16SrIX group phytoplasmas determined by qPCR in plant and insect samples tested in this study.

Sample Locations	Plant				Insect	
	16SrII		16SrIX		16SrII	16SrIX
	Abs.Q^1^	Rel.Q^2^	Abs. Q	Rel.Q	Abs. Q	Abs.Q
Aksu	2.03 × 10^6^	406.48	ND	ND	ND	ND
Aspendos	7.79 × 10^6^	2962.01	8.54 × 10^4^	29.65	ND	ND
Batem	4.37 × 10^6^	3066.46	2.31 × 10^6^	1249.37	7.12 × 10^3^	ND
Belkıs	1.61 × 10^6^	97.5	ND	ND	ND	ND
Beşkonak	ND	ND	ND	ND	ND	ND
Boğazkent	1.12 × 10^6^	871.51	8.84 × 10^4^	221.37	3.14 × 10^4^	ND
Bozova	ND	ND	ND	ND	ND	ND
Cumalı	ND	ND	ND	ND	ND	ND
Denizyaka	6.30 × 10^6^	1374.04	1.80 × 10^5^	142.57	4.26 × 10^4^	ND
Gündoğdu	1.75 × 10^7^	12018.44	8.43 × 10^3^	2.34	ND	ND
Kadriye	2.45 × 10^6^	2134.97	ND	ND	ND	ND
Kocayatak	ND	ND	ND	ND	8.89 × 10^3^	ND
Kovanlık	3.51 × 10^6^	3916.13	5.90 × 10^5^	333.4	6.62 × 10^5^	2.58 × 10^4^
Taşağıl	ND	ND	ND	ND	4.97 × 10^3^	ND
Greenhouse	ND	ND	ND	ND	1.63 × 10^7^	ND
Calibrator ^3^		1		1		

^1^ Shows the absolute quantities of phytoplasma DNA as copy number per milligram (mg) of plant and insect tissue.

^2^ Shows the relative quantities of phytoplasma DNA normalized to sesame DNA and expressed as fold differences relative to a designated calibrator.

^3^ Sesame DNA from uninfected sesame containing the lowest amounts of 16SrII and 16SrIX phytoplasma DNA dilutions (1.8 × 10^2^ and 1.6 × 10^2^ copies for 16SrII and 16SrIX, respectively) per reaction as determined in the standard curve generation in inhibition assays.

ND: Not detected.

Quantities of 16SrII DNA were also found to be quite low in insect samples. Of all districts sampled, insect samples from Kovanlık had the highest quantities of 16SrII DNA (6.62 × 10^5^) per mg. Samples from other districts contained 16SrII DNA ranging from 4.26 × 10^4^ to 4.97 × 10^3^ copies of DNA per mg insect tissue. Insect samples from the campus greenhouse had high 16SrII DNA quantities (1.63 × 10^7^ copies per mg of insect tissue) because these insects were continuously fed on sesame plants with phyllody disease in cages for transmission assays.

Relative quantities of 16SrII and 16SrIX phytoplasmas in the samples generally followed the same trend as absolute quantities. The results from absolute and relative quantifications were checked for consistency by correlation analyses. Relative quantifications and absolute DNA quantities for samples were highly correlated with correlation coefficients of 0.92 and 0.99 for 16SrII and 16SrIX, respectively. This result further indicated the robustness of the qPCR assay.

## Discussion

Phyllody is one of the most economically important and destructive diseases of sesame caused by mollicute phytoplasmas. Here, a sensitive and efficient multiplex qPCR assay was developed to detect, identify, and quantify two phytoplasma groups in sesame and their insect vector. qPCR assay with the universal phytoplasma specific primer pair and 16SrII and 16SrIX group specific probes clearly distinguished these two phytoplasmas from each other and from phytoplasmas belonging to four 16Sr groups. Moreover, a comparison based on the sequence and BLAST analysis indicated that, qPCR universal primers had the power to amplify all 14 phytoplasma groups and the qPCR probes to hybridize only to their respective 16SrII and 16SrIX target groups (S1 Fig). To our knowledge, phytoplasma specific universal primers from 16S ribosomal RNA gene region have not been developed for any phytoplasma-plant systems for qPCR despite the existence of group specific primers [25,29,32–36,52,53]. Furthermore, the probes developed in this study are specific to 16SrII and 16SrIX phytoplasma groups in multiplex assay.

The qPCR assay was proved to be quite sensitive and efficient for detection and quantification of 16SrII and 16SrIX phytoplasmas. Sensitive detection of phytoplasmas through multiplex real-time PCR has been reported for several crops [34,52,53]. In this study, the sensitivity of the multiplex qPCR assay was as low as 1.8 and 1.6 × 10^2^ copies per reaction of 16SrII and 16SrIX phytoplasma cells, respectively. This high level of sensitivity for both phytoplasma groups has been rarely reached in previous studies with other phytoplasma groups [32–35]. Likewise, the standard curve assays demonstrated that sesame DNA amplification by the internal control SEPL-18SDNA-F/R primer pair is highly specific and efficient, allowing for the calculation of relative quantities of both group phytoplasmas. Here, the high sensitivity reached with this assay should facilitate the detection of low levels of 16SrII and 16SrIX group phytoplasmas in sesame plant and insect tissues.

The inhibition assay results further confirmed that multiplex real-time qPCR assay was sensitive and specific to target phytoplasmas and the presence of sesame DNA did not have an inhibitory effect on amplification. DNA extraction without PCR inhibitors is crucial to determine the precise quantities of phytoplasma DNA in infected tissues, since they may cause the complete failure of the multiplex real-time PCR assay or lower the threshold of detection [54]. In the multiplex qPCR, amplification of 16SrII and 16SrIX phytoplasma DNA was shown to have similar efficiency in the presence of sesame DNA and in molecular grade water. Moreover, the standard curves generated by the assay were effective to calculate quantities of 16SrII and 16SrIX DNA in field samples of infected plant and insect tissue.

Absolute and relative qPCR quantification revealed that most of the sesame plant and insect samples from a majority of the 15 locations were infected only with 16SrII group phytoplasmas. However, some samples were only infected with 16SrIX group phytoplasma, while a few had co-infections of both phytoplasma groups. The results from real-time qPCR were in agreement with disease status of field collected plant samples. These results are also consistent with our previous report in which five locations in common with this study were tested through nested PCR and absolute quantities of the 16SrII and 16SrIX DNA were reported to be highly correlated with relative quantities in the infected sesame samples from different locations [39]. However, relative qPCR is more suitable for elucidating the interactions among phytoplasmas and sesame plants and their vectors, and is thus more suitable for comparison of resistance reactions of sesame varieties to phytoplasmas in the field. Hence, the assay will be a useful tool to determine infection levels and disease reaction types of different sesame varieties for which visual observations and ratings are difficult.

Selective breeding for resistance traits is considered to be the most effective method to manage plant diseases in fields [55]. To manage phyllody and related diseases effectively, new sources of sesame resistance must be found and incorporated into commercial varieties. Methods for accurate and sensitive detection and quantification of phytoplasmas in plant tissues are critical to screen and select new sources of germplasm with resistance to these pathogens [56]. In preliminary tests, we have observed resistant and susceptible sesame genotypes showing low and high phytoplasma titers as measured through qPCR developed in this study. The qPCR assay to quantify 16SrII and 16SrIX group phytoplasmas will be a great complement to the visual assays for accurate and quick evaluation of sesame varieties resistant to different phytoplasma pathogens.

## Supporting Information

S1 Fig Sequence alignment of the16S ribosomal gene region used for specific amplification and detection of sesame 16Sr group II and IX phytoplasmas with other 16Sr group phytoplasmas. Sequence regions of the primers designed in this study are shown in red color. The probes specific to 16SrII and 16SrIX are indicated in blue and green color, respectively.
(DOCX)

S2 Fig SYBR^®^ Green real-time PCR of six different phytoplasma groups with universal phytoplasma (A) and housekeeping (B) primers. (DOCX)

S3 Fig Specifity of qPCR assay on six different phytoplasma groups with universal phytoplasma and housekeeping primers. Specific detection of 16SrII (A) and 16SrIX (B) group phytoplasmas and housekeeping gene (C).
(DOCX)
