# Supplementary material for: Multiplex Real-Time qPCR Assay for Simultaneous and Sensitive Detection of Phytoplasmas in Sesame Plants and Insect Vectors
Source: PLoS One. 2016 May 19;11(5):e0155891. doi: 10.1371/journal.pone.0155891 (PMC4873013; doi:10.1371/journal.pone.0155891)
Supplement: S1 Fig — Sequence regions of the primers designed in this study are shown in red color. The probes specific to 16SrII and 16SrIX are indicated in blue and green color, respectively. (DOCX) [file pone.0155891.s001.docx]

540 600

16SrI GCAAGCGTTATCCGGAATT**ATTGGGCGTAAAGGGTGCGTAG**GCTGTTAAATAAGTTTATG

16SrII GCAAGTGTTATCCGGAATT**ATTGGGCGTAAAGGGTGCGTAG**GCGGTCTAGTAAGTCAGTG

16SrIII GCGAGCGTTATCCGGAATT**ATTGGGCGTAAAGGGTGCGTAG**GCGGTTTAATAAGTCTATA

16SrIV GCGAGCGTTATCCGGAATT**ATTGGGCGTAAAGGGTGCGTAG**GCGGTTTAATAAGTCTCTA

16SrV GCGAGCGTTATCCGGAATT**ATTGGGCGTAAAGGGTGCGTAG**GCGGTTAGATAAGTCTATA

16SrVI GCGAGCGTTATCCGGAATT**ATTGGGCGTAAAGGGTGCGTAG**GCTGTTAGATAAGTCTATA

16SrVII GCGAGCGTTATCCGGAATT**ATTGGGCGTAAAGGGTGCGTAG**GCGGTTAGGAAAGTCTATA

16SrVIII GCGAGCGTTATCCGGAATT**ATTGGGCGTAAAGGGTGCGTAG**GCGGTTAGTTAAGTCTATA

16SrIX GCGAGCGTTATCCGGAATT**ATTGGGCGTAAAGGGTGCGTAG**GCGGT**TTGATAAGTCTATA**

16SrX GCAAGCGTTATCCGGATTT**ATTGGGCGTAAAGGGTGTGTAG**GCGGTTTAATAAGTCTATG

16SrXI GCGAGCGTTATCCGGAATT**ATTGGGCGTAAAGGGTGCGTAG**GCGGTTTAATAAGTCTATA

16SrXII GCAAGCGTTATCCGGAATT**ATTGGGCGTAAAGGGTGCGTAG**GCGGTTAAATAAGTTTATG

16SrXIII GCGAGCGTTATCCGGAATT**ATTGGGCGTAAAGGGTGCGTAG**GCGGTTTAATAAGTTTATG

16SrXIV GCGAGCGTTATCCGGAATT**ATTGGGCGTAAAGGGTGCGTAG**GCGGTTTGGTAAGTCTATA

601 660

16SrI GTCTAAGTGCAATGCTCAACATTGTGATGCTATAAAAACTGTTT-AGCTAGAGTAAGATA

16SrII GTGTAATGGCA**ACGCTTAACGTTGTCCGGCTATTGAAACTGCT**A-AACTTGAGTTAGATA

16SrIII GTTTAATTTCAGTGCTTAACGCTGTTGTGCTATAGAAACTGTTT-TACTAGAGTGAGATA

16SrIV GTTTAATTTCAACGCTTAACGTTGTCCTGCTAGAGAAACTGTTT-AACTAGAGTGAGATA

16SrV ATTTAATTTCAGTGCTTAACGCTGTCTTGTTATAGAAACTGTCTTGACTAGAGTGAGATA

16SrVI ATTTAATTTCAGTGCTTAACGCTGTCTTGTTATAGAAACTGTCTTGACTAGAGTGAGATA

16SrVII ATTTAATTTCAGTGCTTAACGCTGTCTTGTTATAGAAACTACCTTGACTAGAGTTAGATA

16SrVIII ATTTAATTTCAACGCTTAACGTTGTTTTGTTATAGAAACTGCCT-AACTAGAGTGAGATA

16SrIX **GTTTAAATGCAGTGCTTAACGC**TGTAGCGCTATAGAAACTGTCT-GACTAGAGTTAGATA

16SrX GTATAAGTTCAACGCTTAACGTTGTGATGCTATGGAAACTGTTT-GACTAGAGTTGGATA

16SrXI GTTTAATTTCAGTGCTTAACACTGTCCTGCTATAGAAACTATTA-GACTAGAGTGAGATA

16SrXII GTCTAAGTGCAACG-TCAACGTTGTGATGCTATAAAAACTGTTT-AGCTAGAGTTGGATA

16SrXIII GTCTAAGTGCAATGCTTAACATTGTGATGCTATAAAAACTGTTT-GACTAGAGTTGGATA

16SrXIV GTTTAATTTCAGTGCTTAACACTGTTCTGCTATAGAAACTATCA-GACTAGAGTGAGATA

661 720

16SrI GAGGCAAGT**GGAATTCCATGTGTAGTGGTAAAATG**CGTA

16SrII GAGGCGAGT**GGAATTCCATGTGTAGCGGTAAAATG**CGTA

16SrIII GAGGCAAGC**GGAATTCCATGTGTAGCGGTAAAATG**CGTA

16SrIV GAGGTAAGC**GGAATTCCATGTGTAGCGGTAAAATG**TGTA

16SrV GAGGCAAGC**GGAATTCCATGTGTAGCGGTAAAATG**TGTA

16SrVI GAGGCAAGC**GGAATTCCATGTGTAGCGGTAAAATG**TGTA

16SrVII GAGGCAAGC**GGAATTCCATGTGTAGCGGTAAAATG**TGTA

16SrVIII GAGGCAAGT**GGAATTCCATGTGTAGCGGTAAAATG**TGTA

16SrIX GAGGCAAGC**GGAATTCCATGTGTAGCGGTAAAATG**CGTA

16SrX GAGGCAAGT**GGAATTCCATGTGTAGCGGTAAAATG**CGTA

16SrXI GAGGCAAGT**GGAATTCCATGTGTAGCGGTAAAATG**CGTA

16SrXII GAGGCAAGT**GGAATTCCGTGTGTAGTGGTAAAATG**CGTA

16SrXIII GAGGCAAGT**GGAATTCCATGTGTAGTGGTAAAATG**CGTA

16SrXIV GAGGCAAGT**GGAATTCCATGTGTAGCGGTAAAATG**CGTA
