# Supplementary material for: Multiplex Real-Time qPCR Assay for Simultaneous and Sensitive Detection of Phytoplasmas in Sesame Plants and Insect Vectors
Source: PLoS One. 2016 May 19;11(5):e0155891. doi: 10.1371/journal.pone.0155891 (PMC4873013; doi:10.1371/journal.pone.0155891)
Supplement: Supplementary file 2 [file pone.0155891.s002.docx]

Cycle number (Ct)

Fluorescence

16SrX-->

<--16SrIII

16SrI-->

<--16SrIX

16SrVII-->

<--16SrII

Neg.Cont.-->

A

Cycle number (Ct)

Fluorescence

16SrIII-->

<--16SrI

16SrX-->

<--16SrII

16SrVII-->

Neg.Cont.-->

16SrIX-->

B
