# Supplementary material for: Multiplex Real-Time qPCR Assay for Simultaneous and Sensitive Detection of Phytoplasmas in Sesame Plants and Insect Vectors
Source: PLoS One. 2016 May 19;11(5):e0155891. doi: 10.1371/journal.pone.0155891 (PMC4873013; doi:10.1371/journal.pone.0155891)
Supplement: Supplementary file 3 [file pone.0155891.s003.docx]

Cycle number (Ct)

Fluorescence

16SrII-->

16SrI

16SrIII

16SrVII

16SrIX

16SrX

Neg.Cont.

A

Cycle number (Ct)

Fluorescence

16SrIX-->

16SrI

16SrII

16SrIII

16SrVII

16SrX

Neg.Cont.

B

Cycle number (Ct)

Fluorescence

Neg.Cont.-->

16SrIII-->

16SrX-->

<--16SrI

16SrVII-->

16SrII-->

16SrIX-->

C
